# Positive Correlation Between Serum Limonene Levels and Muscle Health in a Representative Adult Population in the United States

**DOI:** 10.3390/ph18010074

**Published:** 2025-01-10

**Authors:** Chang-Chin Wu, Yu-Wei Fang, Chikang Wang, Chien-Yu Lin

**Affiliations:** 1 Department of Orthopedics, En Chu Kong Hospital, New Taipei City 237, Taiwan; 00709@km.eck.org.tw; 2 Department of Biomedical Engineering, Yuanpei University of Medical Technology, Hsinchu 300, Taiwan; 3 Department of Orthopaedic Surgery, School of Medicine, National Taiwan University, Taipei 106, Taiwan; 4 Division of Nephrology, Department of Internal Medicine, Shin-Kong Wu Ho-Su Memorial Hospital, Taipei 111, Taiwan; m005916@ms.skh.org.tw; 5 School of Medicine, College of Medicine, Fu Jen Catholic University, New Taipei City 242, Taiwan; 6 Department of Environmental Engineering and Health, Yuanpei University of Medical Technology, Hsinchu 300, Taiwan; ckwang@mail.ypu.edu.tw; 7 Department of Internal Medicine, En Chu Kong Hospital, No. 399, Fuxing Rd., Sanxia Dist., New Taipei City 237, Taiwan

**Keywords:** monoterpenes, hand grip strength, lean muscle mass, limonene, National Health and Nutrition Examination Survey (NHANES)

## Abstract

**Background/Objectives:** Monoterpenes, a class of organic compounds with the molecular formula C_10_H_16_, have garnered significant attention for their potential medicinal benefits. Emerging evidence suggests they may positively influence skeletal muscle function. However, the impact of monoterpene exposure on muscle strength and mass in humans remains unclear. **Methods**: To explore this relationship, we analyzed data from 1202 adults (aged ≥ 18 years) who participated in the 2013–2014 National Health and Nutrition Examination Survey (NHANES), focusing on serum levels of three specific monoterpenes—α-pinene, β-pinene, and limonene—and their association with hand grip strength and lean muscle mass. **Results:** Our analysis revealed that, except for test 2 of hand 1, all grip strength measures showed a positive correlation with ln-limonene levels. The β coefficient for combined grip strength was 2.409 (S.E. = 0.891, *p* = 0.015). Positive associations were also found between serum limonene levels and lean muscle mass. The β coefficient for the Appendicular Skeletal Muscle Mass Index (ASMI) was 0.138 (S.E. = 0.041, *p* = 0.004). Furthermore, combined grip strength and ASMI significantly increased across limonene quintiles (*p* for trend = 0.005 and 0.006, respectively). However, none of the three monoterpene levels showed a significant association with clinically defined low muscle mass or low muscle strength. **Conclusions:** Our findings suggest a plausible association between exposure to limonene, hand grip strength, and lean muscle mass among adults in the United States. Further investigation is needed to fully understand the underlying mechanisms and medical significance of this association.

## 1. Introduction

Monoterpenes, known by their chemical formula C_10_H_16_, encompass a wide range of naturally occurring organic compounds and serve a variety of purposes in essential oils, culinary products, cleaning agents, and beauty items [1]. Humans can come into contact with monoterpenes through inhalation, ingestion, and skin contact [2]. Following exposure, monoterpenes undergo first-phase metabolism, resulting in the production of more water-soluble byproducts that are excreted in urine within a timeframe of 12 to 24 h [3]. The therapeutic effects of monoterpenes have been extensively researched, revealing a wide range of health benefits, including inflammation reduction, antioxidant, pain relief, anticancer, diabetes regulation, and antiviral effects [4]. However, it is important to recognize that recent studies have highlighted possible risks associated with essential oils with high concentrations of monoterpenes, which may lead to neurotoxic, genotoxic, or teratogenic effects [5].

Muscle wasting involves a decrease in both muscle size and strength, resulting in decreased endurance, flexibility, and overall physical performance [6]. Mitigating muscle wasting requires a combination of adequate protein intake, physical activity, and identification of contributing factors. However, there are currently no effective pharmaceutical interventions to reverse muscle wasting [7,8]. Studies suggest that mitochondrial dysfunction and reactive oxygen species (ROS) production play a significant role in the progression of muscle wasting [9]. Recent research has drawn attention to the potential benefits of a diet rich in fruits and vegetables for maintaining muscle health and reducing the risk of sarcopenia in humans [10,11]. Since the perception of taste and aroma in vegetables and fruits is based on the primary constituents known as monoterpenes, their potential effects on skeletal muscle health have been investigated in animal models. One animal study showed that limonene, a commonly used monoterpene, reduced markers of oxidative stress and enzyme activity associated with muscle injury. In addition, a combination treatment with therapeutic ultrasound showed promising results in further reducing oxidative stress [12]. Another monoterpene, camphene, has also shown potential in ameliorating skeletal muscle atrophy by targeting oxidative stress [8]. However, the existing body of research on the potential relationship between exposure to monoterpenes and skeletal muscle well-being in the general population remains unknown.

Dynamometry is a dependable, credible, and sensitive technique for assessing hand grip strength [13]. Furthermore, dual-energy X-ray absorptiometry (DXA) is broadly acknowledged as the benchmark for determining body composition. Assessing lean body mass while excluding bone mineral content serves as a valuable metric for evaluating lean muscle mass [14]. National Health and Nutrition Examination Survey (NHANES) recently incorporated α-pinene, β-pinene, and limonene into biomonitoring efforts due to their extensive environmental exposure, high bioavailability, and potential health implications. These monoterpenes are among the most abundant volatile organic compounds emitted by vegetation, particularly coniferous trees, and are frequently found in both indoor and outdoor environments from sources such as air fresheners, cleaning products, and plant-based resins [15]. To bridge this knowledge gap, we examined data from the NHANES conducted during the period of 2013–2014. This dataset provides information on three serum levels of monoterpenes (α-pinene, β-pinene, and limonene), hand grip strength assessments using dynamometry, and measurements of lean muscle mass via DXA in our present investigation. Our study sought to augment our understanding of the link between monoterpene levels and muscle health within the general adult population of the United States.

## 2. Results

The study enrolled a total of 1202 participants, with a mean age of 46.86 years and a median age of 46.00 years. The age range of the participants spanned from 18 to 80 years. The mean BMI was 29.15 kg/m^2^, with a range spanning from 17.42 to 62.10 kg/m^2^ and a standard deviation of 7.11. The mean concentrations of α-pinene, β-pinene, and limonene, along with their respective standard deviations, were 0.095 ng/mL (0.068), 0.095 ng/mL (0.080), and 1.470 ng/mL (1.190). The detection rates for these three chemicals were 74.3%, 74.6%, and 100%, respectively. In the analysis, the natural logarithm of the three monoterpenes and creatinine was used to account for their non-Gaussian distributions. Notably, there was a significant correlation observed between the concentrations of the three monoterpenes, with ln-α-pinene and ln-β-pinene exhibiting the highest correlation, as indicated by Spearman’s correlation coefficient of 0.754 (*p* < 0.001). In Table 1, the study presents the average values of the three monoterpenes across different subgroups. The findings from the study suggest that certain factors influence the levels of these monoterpenes. Men and current smokers displayed higher levels of limonene, while individuals with a higher BMI exhibited elevated levels of α-pinene and β-pinene. Additionally, Mexican-Americans demonstrated higher levels of β-pinene and limonene.

**Figure 1:**
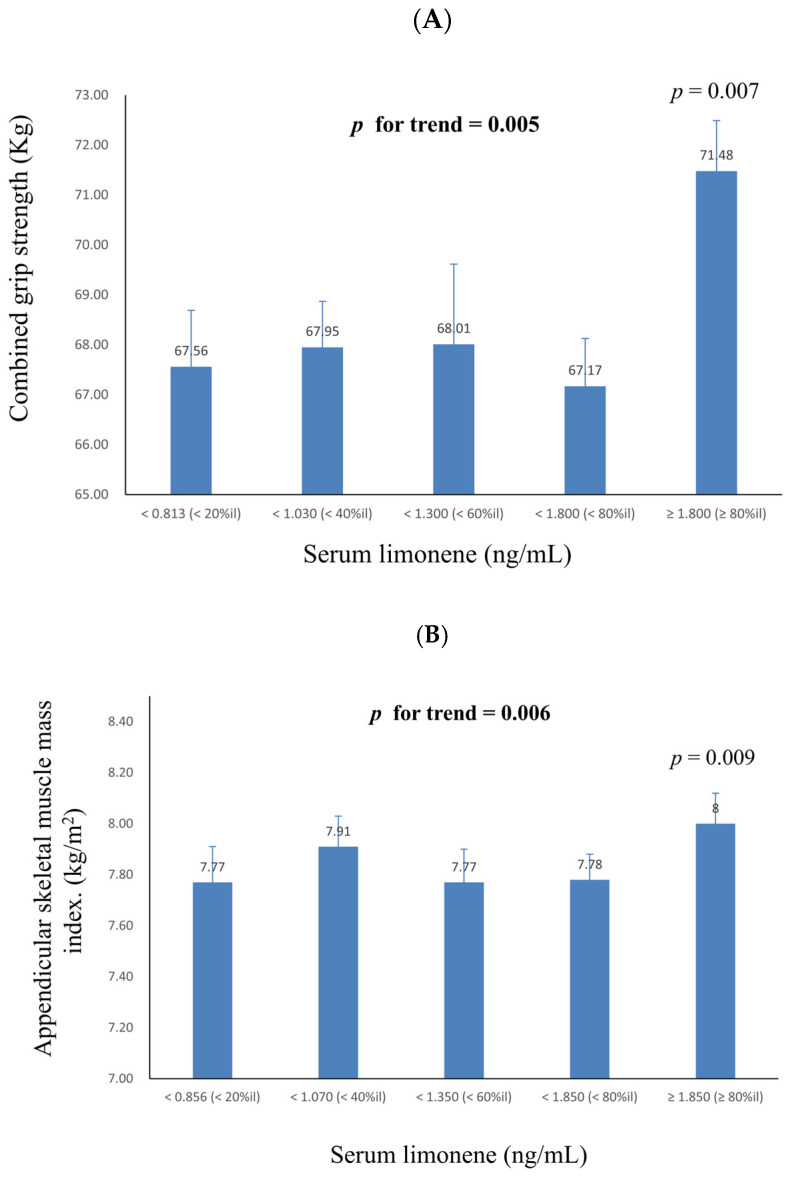
Hand grip strength and ASMI across quintiles of serum limonene in multiple linear regression models, with results weighted for sample strategy. (**A**): Combined grip strength (n = 1168). (**B**): Appendicular Skeletal Muscle Mass Index (n = 754). Results represent adjusted means, with comparisons made against the lowest quintile of serum limonene (Q1). Statistically significant differences (*p* < 0.05) are indicated.

Table 2 provides coefficients obtained from linear regression analysis, highlighting the relationship between grip strength and a one-unit increase in ln-serum monoterpenes. The ß coefficient represents the expected change in grip strength associated with a one-unit increase in ln-monoterpenes levels, holding other variables constant. This allows for a standardized interpretation of the strength and direction of the relationship. Notably, all grip strength measurements, except for test 2 of hand 1, displayed a positive correlation with ln-limonene levels. For combined grip strength, the ß coefficient was calculated as 2.409 (S.E. = 0.891, *p* = 0.015). In Table 3, the study presents the linear regression coefficients for lean muscle mass and a one-unit increase in ln-serum monoterpenes. Positive associations were observed between limonene levels and lean muscle mass in various areas, including both arms and legs, trunk, total body, and ASMI. Specifically, the β coefficient for ASMI was 0.138 (S.E. = 0.041, *p* = 0.004). Figure 1 offers a graphical representation of the relationship between combined grip strength and ASMI across serum limonene quintiles in multiple linear regression models. The results indicate a significant increase in both combined grip strength and ASMI as the limonene quintiles increase (*p* for trend = 0.005 and 0.006, respectively). Furthermore, when compared to the lowest limonene quintile, both combined grip strength and ASMI at the highest limonene quintile showed a significant increase (*p* = 0.007 and 0.009, respectively).

Table 4 presents the odds ratios of low muscle mass or low muscle strength associated with a one-unit increase in ln-monoterpenes, as determined by logistic regression models. However, none of these three monoterpene levels exhibit statistical significance in relation to the risk of low muscle mass or low muscle strength. Lastly, Table 5 delves into the connection between limonene and combined grip strength as well as ASMI across diverse subgroups of study participants. The link between combined grip strength and limonene was remarkably robust within specific subgroups, including men, individuals of non-Hispanic white ethnicity, those with lower income, and individuals with a BMI ranging from 25 to 30. Additionally, the study observed significant interactions between gender and limonene, as well as between BMI and limonene, concerning their association with combined grip strength. Regarding the relationship between ASMI and limonene, it exhibited particular significance among men, younger individuals, ethnic groups other than non-Hispanic white, individuals with lower income, and those with a higher BMI. Importantly, no significant interaction was identified between subgroups and limonene concerning its impact on ASMI.

## 3. Discussion

Our investigation utilized a representative sample of the adult population and revealed a substantial positive association between serum concentrations of limonene, hand grip strength, and lean muscle mass. In addition, an interaction between limonene and sex and limonene and BMI was identified with respect to the association with combined grip strength. Despite the fact that none of these three monoterpene concentrations reached statistical significance in relation to the risk of low muscle strength or low muscle mass, this study provides preliminary evidence of a possible association between exposure to limonene and overall muscle health in the general adult population. The significance of this study stems from the reliable and comprehensive data collected from the NHANES database, as well as the inclusion of a representative sample of American adults, which ensures its broader applicability.

The findings from our study suggest that limonene demonstrates a greater frequency and magnitude of exposure in comparison to the other two monoterpenes. Furthermore, we observed increased levels of limonene in males and individuals who are currently smokers. Among the monoterpenes, limonene is widely employed as a flavor enhancer in the food industry and is recognized as a significant ingredient in cosmetic and household products [16,17]. Moreover, introducing monoterpene synthases from lemon into genetically modified tobacco plants results in elevated levels of limonene when compared to the natural variant [18]. These findings provide evidence that supports the notion of increased internal exposure to limonene relative to the other two monoterpenes. In addition, we noted heightened levels of β-pinene and limonene among individuals of Asian heritage, along with increased levels of β-pinene in those with a higher BMI. These variances could potentially be linked to disparities in dietary habits, cultural practices, and genetic predisposition. To gain a comprehensive understanding of the factors underlying the variations in monoterpene levels among different subpopulations, further research and investigation are warranted.

Humans can come into contact with monoterpenes through inhalation, ingestion, and skin contact [2]. A previous investigation has indicated that children at daycare centers are primarily exposed to monoterpenes through their diet and consumer goods, rather than through respiratory means [19]. Recent studies have attempted to measure the presence of monoterpenes in the diet, but the considerable variation in their content poses challenges when estimating human intake [3]. Exclusively concentrating on the serum levels of monoterpenes, the NHANES 2013–2014 database reveals a significant detection rate, emphasizing the inevitability of exposure to monoterpenes during daily activities. These results highlight the need for additional investigations into the origins of monoterpenes, specifically among the general population.

Earlier experimental research has investigated the potential effects of administering monoterpenes to muscle cells. The effects of camphene, a bicyclic monoterpene, on muscle atrophy were examined through in vitro and in vivo investigations. In studies conducted on rat skeletal muscle cells, camphene demonstrated the ability to reverse abnormal muscle cell morphology, decrease indicators of oxidative damage, and regulate lipid metabolism. Additionally, in Sprague Dawley rat models, camphene exhibited significant regulation of lipid metabolism in cells treated with H_2_O_2_ [8]. In another study using male Wistar rats, the effects of topical limonene, alone or combined with therapeutic ultrasound, on oxidative stress caused by gastrocnemius muscle injury were investigated. The findings revealed that therapeutic ultrasound alone, topical limonene application, and phonophoresis with limonene effectively reduced lipid peroxidation levels and superoxide dismutase activity following the muscle injury. Specifically, only the combination of phonophoresis with limonene reduced creatine kinase activity after the injury [12]. It is worth noting that the majority of research on α-pinene and β-pinene has focused on their effects on other physiological systems. As a result, the direct effects of these compounds on skeletal muscles, including their antioxidant activity, have not been well-documented in scientific literature [20].

Consuming vegetables and fruits, known for their abundance of vitamins, minerals, phytochemicals, and dietary fiber, is considered crucial for maintaining a healthy diet, and their intake is associated with numerous positive health benefits [10]. One epidemiologic study in Korea indicated that older adults who consume fewer fruits and vegetables are more likely to experience sarcopenia, particularly in men [21]. Furthermore, an eating pattern encompassing vegetables and fruits has been linked to reduced likelihood of sarcopenia among elderly Chinese men [11]. Moreover, a recent small-scale randomized controlled trial conducted in the UK revealed a tendency towards enhanced grip strength among a group that consumed higher quantities of fruits and vegetables [22]. The results underscore the potential advantages of adopting a diet abundant in vegetables and fruits for preserving muscle health and reducing the risk of sarcopenia among older individuals. Since the perception of taste and aroma in vegetables and fruits relies on monoterpenes as their primary constituents, the benefit health effects of the monoterpenes are based on animal studies rather than clinical studies [3]. Due to the scarcity of previous studies examining the link between exposure to monoterpenes and skeletal muscle strength/mass in the general population, our research provided the first evidence of a positive correlation between the concentration of limonene in the bloodstream and both hand grip strength and lean muscle mass among adult individuals in the United States. Furthermore, our findings indicate a clear dose–response relationship, as higher levels of limonene were associated with a significant increase in both combined grip strength and ASMI.

Since ROS generation and energy metabolism are key mechanisms for muscle atrophy [23], limonene has been extensively studied for its antioxidant properties and has demonstrated significant antioxidant activity in various studies. In an animal study, Sprague Dawley rats were orally administered d-limonene at doses of 50 mg/kg and 100 mg/kg. The result found that d-limonene administration significantly increased the activities of superoxide dismutase by up to 66.48%. Additionally, it raised the levels of reduced glutathione by up to 72.96% when given at the highest dosage [24]. In addition to its antioxidant properties, limonene may also exert anti-inflammatory effects that contribute to its potential role in improving muscle health. Chronic low-grade inflammation has been recognized as a key factor in the development and progression of sarcopenia, as it promotes catabolic processes and impairs anabolic signaling pathways in skeletal muscles [25]. Limonene has demonstrated significant anti-inflammatory activity in various experimental models. For instance, limonene has been shown to inhibit the production of pro-inflammatory cytokines such as TNF-α, IL-6, and IL-1β, as well as reduce the activation of nuclear factor kappa B, a critical regulator of inflammation [26]. Given that ROS generation and inflammation are closely interconnected and jointly contribute to muscle atrophy, limonene’s dual actions as an antioxidant and anti-inflammatory agent may synergistically protect against muscle damage and enhance muscle function. Future studies should aim to elucidate the molecular pathways by which limonene modulates oxidative stress and inflammation in skeletal muscle.

We do not observe an association between the other two monoterpenes (α-pinene and β-pinene) and muscle strength and mass. This discrepancy may stem from structural and functional differences among these monoterpenes, which can influence their biological activity, bioavailability, and interactions with cellular pathways. Limonene possesses two conjugated double bonds in its chemical structure [27], which are known to enhance its antioxidant capacity by stabilizing free radicals more effectively [28]. In contrast, α-pinene and β-pinene each have only one double bond, potentially limiting their antioxidant potential compared to limonene [4]. The stronger antioxidant activity of limonene might contribute more effectively to reducing oxidative stress in skeletal muscle, which is a key mechanism underlying muscle atrophy and dysfunction. Another possible explanation could be differences in bioavailability and metabolism of these compounds in humans. Limonene is metabolized into bioactive compounds, such as perillic acid and carveol, which may exert additional biological effects on muscle tissues [29]. In contrast, the metabolic derivatives of α-pinene and β-pinene may not exhibit similar muscle-protective effects. Future research should aim to further explore the specific molecular pathways through which these monoterpenes exert their effects and investigate whether differences in their metabolism and tissue distribution might explain the observed variability in their associations with muscle health outcomes.

Our study uncovered a notable interaction between limonene and sex, and limonene and BMI, in terms of their correlation with combined grip strength. This interaction could be due to differences in genetics, lifestyle, or hormone levels between men and women. In addition, individuals with a higher BMI, who are already at increased risk for inflammation and oxidative stress-related diseases, may experience a reduced effect. Based on our current knowledge, this study represents the first instance in which specific demographic subgroups have been identified as potentially susceptible to the beneficial effects of limonene on hand grip strength. Additional research is needed to fully understand the potential mechanisms underlying these observed differences.

The clinical implications of our findings suggest that limonene, a naturally occurring monoterpene commonly found in citrus fruits, may play a role in maintaining skeletal muscle health. Given its positive association with hand grip strength and lean muscle mass observed in our study, limonene could potentially serve as a dietary supplement for promoting muscle health, particularly in populations at risk of sarcopenia, such as older adults. Dietary supplementation with limonene may offer a simple and cost-effective strategy to support muscle strength and mass, especially when combined with other interventions such as resistance exercise and adequate protein intake. Our study highlights limonene as a promising candidate for future clinical research and potentially as a functional dietary ingredient aimed at muscle health preservation.

In evaluating the results, it is important to recognize the limitations of this study. First, the investigation was limited to NHANES 2013–2014 data and focused only on serum monoterpenes, hand grip strength, and lean muscle mass. This limited the number of cases and made it difficult to conduct a comprehensive analysis. Second, due to the cross-sectional design of the study, it is not possible to establish causal relationships. Thirdly, the study did not account for possible coexposure to other compounds associated with monoterpenes. It is plausible that individuals with elevated serum levels of monoterpenes may also have increased exposure to other substances. This factor could potentially shed light on the observed results. Finally, the study focused exclusively on adult participants from the United States, limiting the generalizability of the findings to other age demographics and geographic areas.

## 4. Materials and Methods

### 4.1. Study Population

**Figure 2:**
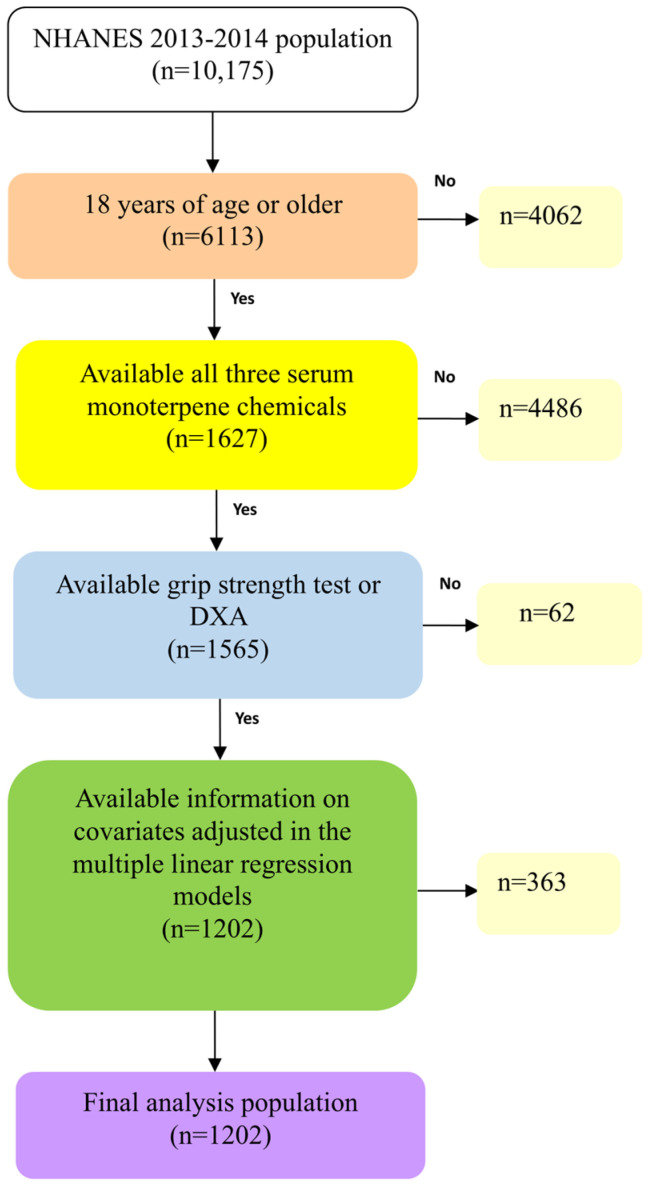
Flow chart algorithm.

NHANES is a national survey conducted every two years to provide a representative sample of the U.S. population. The survey methodology and consent forms are available on the NHANES website [30]. For our research, we used the 2013–2014 NHANES database, which included 10,175 participants. From this dataset, we specifically selected 6113 individuals who were 18 years of age or older. After excluding participants without all three monoterpene chemicals, the remaining number was 1627. We also excluded 62 participants with incomplete data for the grip strength test or DXA. From the initial sample of 1565 subjects, we further excluded 363 individuals due to missing covariates, which were adjusted in the multiple linear regression. Finally, our analysis focused on a sample size of 1202 individuals. A flowchart of the algorithm is shown in Figure 2.

### 4.2. Measurement of Serum Monoterpene Levels

A subset of participants aged 6 years and older within the NHANES 2013–2014 study had three specific monoterpenes (α-pinene, β-pinene, and limonene), which accounted for approximately one-third of the total sample size. The measurement methods for these monoterpenes in the NHANES 2013–2014 analysis were previously documented [15]. In cases where monoterpene levels fell below the limits of detection (LODs), NHANES employed a standard imputation method: undetectable values were replaced with a calculated value equal to the LOD divided by the square root of 2. Our analysis specifically focused on data from individuals aged 18 years and older. For detailed information on the analytical methodology used in the study, please refer to the NHANES website [31].

### 4.3. Measurement of Muscle Strength—Hand Grip Strength Test

In the NHANES 2013–2014 study, the strength of participants’ hand grips was assessed using a dynamometer for individuals aged 6 and above. Participants who had undergone hand or wrist surgery within the past three months or were unable to grip the dynamometer with both hands were excluded. For our study, we collected data from individuals aged 18 and over. During the assessment, participants squeezed the dynamometer as forcefully as possible with one hand, followed by the other hand. Each hand performed three repetitions, with a 60 s rest period between measurements of the same hand. The combined grip strength was calculated by summing the highest readings from each hand. Grip strength cutoff points of less than 30 kg in men and less than 20 kg in women indicate low muscle strength [32]. For more detailed instructions, please refer to the NHANES website [33].

### 4.4. DXA—Lean Muscle Mass

The NHANES DXA scan provides a comprehensive assessment of body composition, covering the entire body. Eligibility for the scan includes individuals between the ages of 8 and 59, with exceptions for pregnant individuals, those who recently received radiographic contrast material, or those exceeding the weight or height limit of the DXA table. In this study, we analyzed data from individuals aged 18 and over to explore the relationship between serum limonene levels and muscle mass in adults. To represent lean muscle mass, we utilized lean body mass as a measurement, specifically excluding bone mineral content. The Appendicular Skeletal Muscle Mass Index (ASMI) was calculated by dividing the total muscle mass of the four limbs by the square of height. Low muscle mass was determined using ASMI values below 7.26 kg/m^2^ for men and below 5.45 kg/m^2^ for women, which were derived from two standard deviations below the average values for healthy young adults aged 18–40 years [34,35]. For detailed information on the examination protocol, please consult the NHANES website [36].

### 4.5. Covariates

Data collection procedures were standardized across all NHANES study sites, as stated on the NHANES website. During household interviews, information on sociodemographic factors such as age, sex, and race/ethnicity was gathered. Participants were categorized based on their responses to the smoking questionnaire, dividing them into active smokers, those exposed to environmental tobacco smoke (ETS), or non-smokers [37]. The alcohol consumption questionnaire determined whether participants had consumed at least 12 alcoholic beverages in the past year, and their responses were categorized accordingly. Total energy and total protein intake calculations involved averaging data from two days of dietary intake questionnaires. Physical activity was assessed by aggregating activity scores and multiplying them by the corresponding metabolic equivalent of task scores, following the guidelines provided on the NHANES website [38]. For this study, chronic kidney disease was defined as an estimated glomerular filtration rate of less than 60 mL/min per 1.73 square meters [39]. Additionally, potential confounding factors such as body mass index (BMI) and diabetes mellitus were taken into account in the analysis.

### 4.6. Statistics

Serum concentrations of three different monoterpenes in different subgroups were presented as geometric mean ± standard error (S.E.). Comparisons between groups were made using two-tailed Student’s *t*-test and one-way analysis of variance to compare geometric means. To assess the correlation between serum monoterpene levels, hand grip strength, and lean muscle mass, a general linear model complex sample was used. We also examined hand grip strength and ASMI across quintiles of serum limonene to assess the potential dose–response relationship. In addition, a complex pattern of logistic regression analysis was conducted to examine the potential association between monoterpene concentrations and the presence of low muscle mass and low muscle strength. Sample weights used followed the protocols specified on the NHANES website [40]. Several covariates were adjusted for, including age, sex, ethnicity, smoking status, alcohol consumption, household income, BMI, physical activity, total energy intake, total protein intake, diabetes mellitus, and chronic kidney disease. Statistical analysis was performed using SPSS version 20 (SPSS Inc., Chicago, IL, USA) with a significance threshold set at two-sided *p* < 0.05.

## 5. Conclusions

In conclusion, we found significant evidence of a positive association between serum levels of limonene, hand grip strength, and lean muscle mass based on our examination of a representative subset of American adults. In addition, we observed an association between limonene and gender, and between limonene and BMI in relation to combined grip strength. Although additional research is needed to determine the clinical significance and causality of our findings, they highlight the need for ongoing studies into the potential beneficial effects of limonene on the musculoskeletal system in adult populations.

## Figures and Tables

**Table 1:** Basic demographics of the sample subjects including geometric means (geometric S.E.) of monoterpene concentrations.

	No.	α-Pinene (ng/mL)	*p* Value	β-Pinene (ng/mL)	*p* Value	Limonene (ng/mL)	*p* Value
Total	1202	0.078 (1.018)		0.078 (1.016)		1.242 (1.015)	
Sex			0.103		0.265		0.006
Men	588	0.081 (1.025)		0.080 (1.023)		1.296 (1.023)	
Women	614	0.076 (1.026)		0.077 (1.023)		1.193 (1.021)	
Age (in years)			0.168		0.071		0.019
18–39	461	0.077 (1.029)		0.079 (1.028)		1.284 (1.026)	
40–59	397	0.082 (1.030)		0.081 (1.028)		1.266 (1.024)	
≥60	344	0.077 (1.033)		0.074 (1.030)		1.161 (1.030)	
Ethnicity			0.514		<0.001		<0.001
Mexican-American	160	0.082 (1.054)		0.095 (1.051)		1.360 (1.041)	
Other Hispanic	99	0.078 (1.067)		0.079 (1.061)		1.191 (1.052)	
Non-Hispanic white	592	0.076 (1.025)		0.073 (1.022)		1.171 (1.020)	
Non-Hispanic black	208	0.083 (1.043)		0.080 (1.039)		1.316 (1.044)	
Non-Hispanic Asian	107	0.078 (1.059)		0.087 (1.069)		1.459 (1.058)	
Other ethnicity	36	0.075 (1.090)		0.074 (1.075)		1.080 (1.094)	
Household income (USD)			0.827		0.783		0.514
<4500	565	0.078 (1.026)		0.079 (1.024)		1.229 (1.022)	
≥4500	637	0.079 (1.024)		0.078 (1.023)		1.254 (1.022)	
BMI (kg/m^2^)			0.007		0.002		0.298
<25	357	0.072 (1.032)		0.072 (1.027)		1.203 (1.028)	
25–30	388	0.080 (1.031)		0.081 (1.030)		1.278 (1.026)	
≥30	457	0.082 (1.029)		0.082 (1.028)		1.242 (1.026)	
Smoking status			0.140		0.115		<0.001
Non-smoker	720	0.076 (1.023)		0.077 (1.021)		1.189 (1.020)	
ETS	207	0.084 (1.044)		0.084 (1.043)		1.240 (1.042)	
Current smoker	275	0.080 (1.036)		0.078 (1.033)		1.393 (1.028)	
Alcohol consumption (drinks/year)			0.525		0.832		0.506
<12	312	0.077 (1.037)		0.079 (1.034)		1.221 (1.029)	
≥12	890	0.079 (1.020)		0.078 (1.019)		1.249 (1.018)	

Abbreviation: BMI, body mass index; ETS, environmental tobacco smoke.

**Table 2:** Linear regression coefficients (standard error) of grip strength with a unit increase in ln-serum monoterpenes in multiple linear regression models, with results weighted for sampling strategy.

Grip Strength (Kg)	Unweighted No.	α-Pinene (ng/mL)		β-Pinene (ng/mL)		Limonene (ng/mL)	
	/Population Size	Adjusted β (S.E.)	*p* Value	Adjusted β (S.E.)	*p* Value	Adjusted β (S.E.)	*p* Value
Hand 1, test 1	1191/178,609,942	0.201 (0.515)	0.701	0.678 (0.472)	0.172	0.854 (0.379)	0.040
Hand 1, test 2	1186/178,155,139	0.230 (0.466)	0.629	0.619 (0.430)	0.171	1.030 (0.491)	0.053
Hand 1, test 3	1186/178,155,139	0.363 (0.496)	0.476	0.773 (0.481)	0.129	1.149 (0.464)	0.026
Hand 1, average	1186/178,155,139	0.269 (0.480)	0.584	0.692 (0.446)	0.142	1.009 (0.424)	0.031
Hand 2, test 1	1170/175,405,074	0.281 (0.441)	0.534	0.590 (0.373)	0.134	1.156 (0.515)	0.040
Hand 2, test 2	1169/175,311,884	0.414 (0.428)	0.348	0.763 (0.377)	0.061	1.407 (0.432)	0.005
Hand 2, test 3	1168/175,242,705	0.366 (0.414)	0.390	0.769 (0.358)	0.049	1.411 (0.471)	0.009
Hand 2, average	1168/175,242,705	0.349 (0.409)	0.408	0.709 (0.353)	0.063	1.329 (0.460)	0.011
Combined grip *	1168/175,242,705	0.751 (0.822)	0.375	1.517 (0.758)	0.064	2.409 (0.891)	0.016

Model adjusted for age, gender, ethnicity, smoking status, drinking status, household income, BMI, physical activity, total energy intake, total protein intake, diabetes mellitus, and chronic kidney disease. Abbreviation: BMI, body mass index. * Combined grip strength: sum of the largest reading from each hand.

**Table 3:** Linear regression coefficients (standard error) of lean muscle mass with a unit increase in ln-serum monoterpenes in multiple linear regression models, with results weighted for sampling strategy.

	Unweighted No.	α-Pinene (ng/mL)		β-Pinene (ng/mL)		Limonene (ng/mL)	
	/Population Size	Adjusted β (S.E.)	*p* Value	Adjusted β (S.E.)	*p* Value	Adjusted β (S.E.)	*p* Value
Lean muscle mass (gm) *							
Right arm	798/122,818,974	38.544 (30.532)	0.226	44.095 (30.112)	0.164	116.900 (28.364)	0.001
Left arm	807/123,629,514	43.745 (31.902)	0.190	51.055 (28.717)	0.096	146.544 (32.800)	<0.001
Right leg	785/120,938,304	−102.970 (82.641)	0.232	−33.051 (77.968)	0.678	191.612 (54.618)	0.003
Left leg	787/120,737,754	−92.168 (80.073)	0.268	−4.098 (63.033)	0.949	260.870 (53.826)	<0.001
Trunk	789/120,403,056	42.331 (154.191)	0.787	193.309 (186.481)	0.316	709.181 (145.878)	<0.001
Total body	758/116,105,506	−177.240 (383.599)	0.651	348.430 (313.828)	0.284	1362.214 (247.565)	<0.001
ASMI (kg/m^2^) **	754/115,701,600	−0.030 (0.058)	0.615	−0.020 (0.056)	0.719	0.138 (0.041)	0.004

Model adjusted for age, gender, ethnicity, smoking status, drinking status, household income, BMI, physical activity, total energy intake, total protein intake, diabetes mellitus, and chronic kidney disease. Abbreviation: BMI, body mass index; ASMI, Appendicular Skeletal Muscle Mass Index. * Lean muscle mass was defined as lean body mass excluding bone mineral content. ** ASMI was defined as the sum of the muscle mass of the 4 limbs divided by height^2^.

**Table 4:** Odds ratios (95% confidence interval (C.I.)) of low muscle mass or low muscle strength with one unit increase in ln-monoterpenes in logistic regression models, with results weighted for sampling strategy.

	Unweighted No.	α-Pinene (ng/mL)		β-Pinene (ng/mL)		Limonene (ng/mL)	
	/Population Size	Odds Ratio (95% C.I.)	*p* Value	Odds Ratio (95% C.I.)	*p* Value	Odds Ratio (95% C.I.)	*p* Value
Low muscle mass *	768/117,696,892	1.181 (0.686–2.031)	0.524	1.134 (0.556–2.312)	0.713	0.929 (0.488–1.769)	0.812
Low muscle strength **	1193/178,804,473	0.929 (0.526–1.641)	0.787	0.826 (0.360–1.899)	0.632	0.889 (0.479–1.650)	0.691

Model adjusted for age, gender, ethnicity, smoking status, drinking status, household income, BMI, physical activity, total energy intake, total protein intake, diabetes mellitus, and chronic kidney disease. Abbreviation: BMI, body mass index. * Low muscle mass was defined as Appendicular Skeletal Muscle Mass Index < 7.26 kg/m^2^ in men < 5.45 kg/ m^2^ in women. ** Low muscle strength was defined as maximal hand grip strength test < 30 kg in men and < 20 kg in women.

**Table 5:** Linear regression coefficients (standard error) of combined grip strength* and ASMI per unit increase in ln-limonene in subpopulation, with results weighted for sampling strategy.

	Combined Grip Strength (kg) *				ASMI (kg/m^2^) **			
	Unweighted No./ Population Size	Adjusted *β* (S.E.)	*p* Value	*p* for Interaction	Unweighted No./ Population Size	Adjusted *β* (S.E.)	*p* Value	*p* for Interaction
Gender				0.030				0.466
Men	574/85,615,956	4.536 (1.475)	0.008		368/58,166,339	0.158 (0.070)	0.039	
Women	594/89,594,587	−0.388 (0.683)	0.579		386/57,535,261	0.092 (0.071)	0.215	
Age (years)				0.389				0.377
18–40	456/66,814,217	2.671 (1.262)	0.051		407/59,214,137	0.207 (0.062)	0.004	
40–59	380/62,012,264	3.291 (1.861)	0.097		347/56,487,463	0.007 (0.090)	0.941	
≥ 60	332/46,384,062	−0.165 (1.657)	0.922					
Ethnicity				0.748				0.471
Non-Hispanic white	576/119,569,720	3.378 (1.107)	0.008		333/72,220,227	0.097 (0.070)	0.187	
Other	592/55,640,823	1.059 (1.092)	0.349		421/43,481,373	0.208 (0.063)	0.005	
Household income (USD)				0.431				0.111
≤4500	539/63,889,106	3.576 (0.881)	0.001		323/39,226,626	0.245 (0.086)	0.012	
>4500	629/111,321,437	1.935 (1.222)	0.134		431/76,474,973	0.094 (0.063)	0.158	
BMI (kg/m^2^)				0.004				0.589
<25	349/51,528,692	2.420 (1.225)	0.067		259/38,522,835	0.078 (0.081)	0.349	
25–30	377/60,821,769	4.533 (1.418)	0.006		230/38,535,119	0.091 (0.070)	0.212	
≥30	442/62,860,083	−0.139 (1.146)	0.905		265/38,643,646	0.190 (0.087)	0.047	

Model adjusted for age, gender, ethnicity, smoking status, drinking status, household income, BMI, physical activity, total energy intake, total protein intake, diabetes mellitus, and chronic kidney disease. Abbreviation: BMI, body mass index. * Combined grip strength: sum of the largest reading from each hand. ** ASMI was defined as the sum of the muscle mass of the 4 limbs divided by height^2^.

## Data Availability

The original data presented in the study are openly available on the NHANES website (https://wwwn.cdc.gov/nchs/nhanes/continuousnhanes/default.aspx?BeginYear=2013 (accessed on 1 January 2025).
